# Amphiregulin-EGFR Signaling Mediates the Migration of Bone Marrow Mesenchymal Progenitors toward PTH-Stimulated Osteoblasts and Osteocytes

**DOI:** 10.1371/journal.pone.0050099

**Published:** 2012-12-31

**Authors:** Ji Zhu, Valerie A. Siclari, Fei Liu, Jordan M. Spatz, Abhishek Chandra, Paola Divieti Pajevic, Ling Qin

**Affiliations:** 1 McKay Orthopaedic Research Laboratory, Department of Orthopaedic Surgery, Perelman School of Medicine, University of Pennsylvania, Philadelphia, Pennsylvania, United States of America; 2 Department of Plastic and Reconstructive Surgery, Shanghai Ninth People's Hospital, Shanghai, China; 3 Endocrine Unit, Massachusetts General Hospital and Harvard Medical School, Boston, Massachusetts, United States of America; Georgia Health Sciences University, United States of America

## Abstract

Intermittent administration of parathyroid hormone (PTH) dramatically increases bone mass and currently is one of the most effective treatments for osteoporosis. However, the detailed mechanisms are still largely unknown. Here we demonstrate that conditioned media from PTH-treated osteoblastic and osteocytic cells contain soluble chemotactic factors for bone marrow mesenchymal progenitors, which express a low amount of PTH receptor (PTH1R) and do not respond to PTH stimulation by increasing cAMP production or migrating toward PTH alone. Conditioned media from PTH-treated osteoblasts elevated phosphorylated Akt and p38MAPK amounts in mesenchymal progenitors and inhibition of these pathways blocked the migration of these progenitors toward conditioned media. Our previous and current studies revealed that PTH stimulates the expression of amphiregulin, an epidermal growth factor (EGF)-like ligand that signals through the EGF receptor (EGFR), in both osteoblasts and osteocytes. Interestingly, conditioned media from PTH-treated osteoblasts increased EGFR phosphorylation in mesenchymal progenitors. Using several different approaches, including inhibitor, neutralizing antibody, and siRNA, we demonstrate that PTH increases the release of amphiregulin from osteoblastic cells, which acts on the EGFRs expressed on mesenchymal progenitors to stimulate the Akt and p38MAPK pathways and subsequently promote their migration in vitro. Furthermore, inactivation of EGFR signaling specifically in osteoprogenitors/osteoblasts attenuated the anabolic actions of PTH on bone formation. Taken together, these results suggest a novel mechanism for the therapeutic effect of PTH on osteoporosis and an important role of EGFR signaling in mediating PTH's anabolic actions on bone.

## Introduction

Osteoporosis is a major public health threat for more than 28 million Americans, affecting mostly postmenopausal women and the elderly. It is a chronic bone disease mainly caused by an imbalance in bone formation by osteoblasts and bone resorption by osteoclasts. While continuous administration of parathyroid hormone (PTH) causes bone loss, current interest in PTH focuses on its ability to strongly augment bone mass in severe osteoporosis patients by intermittent administration [1], [2]. Indeed, Teriparatide, a recombinant form of human PTH 1–34, is the only FDA-approved anabolic treatment for osteoporosis that functions by stimulating bone formation in contrast to most other osteoporosis drugs that suppress bone resorption.

The intact form of PTH is an 84-amino acid polypeptide secreted from the parathyroid glands in response to changes in serum calcium levels. In bone, PTH acts directly on cells of osteoblast lineage (mainly osteoblasts and osteocytes) and indirectly on osteoclasts because only osteoblasts express PTH type I receptor (PTH1R) [3], [4], a transmembrane G-protein coupled receptor. Binding of PTH or PTH-related peptide (PTHrP) to PTH1R activates two well-defined intracellular signal transduction pathways: the protein kinase A (PKA) pathway, in which Gαs stimulates production of cAMP and activation of PKA, and the protein kinase C (PKC) pathway where Gαq activates phospholipase Cβ with subsequent formation of diacylglycerol, PKC activation and formation of 1,4,5-inositol trisphosphate. In osteoblasts, PTH regulates most of its target proteins through the PKA pathway (reviewed in [5]). Previous investigations have identified a number of PTH-responsive genes in osteoblasts. Our microarray studies revealed 125 PTH-regulated genes in osteoblastic UMR 106-01 cells [6] and more than 300 PTH-regulated genes in the rat femoral osteoblast-rich secondary spongiosa after PTH injections [7].

Extensive investigations have been performed to understand the cellular mechanisms by which intermittent injection of PTH increases osteoblast numbers. It has been concluded that, multiple mechanisms, including activating bone lining cells, stimulating osteoblast differentiation from osteoprogenitors, and preventing osteoblast and osteocyte apoptosis, contribute to the anabolic action of PTH [5], [8]. However, whether PTH regulates the bone marrow mesenchymal progenitors, including mesenchymal stem cells, the multipotent progenitors for osteoblasts, chondrocytes and adipocytes, and more committed osteoprogenitors, is not clear. Several studies have investigated the effects of PTH on these cells by assessing the number of colony forming unit-fibroblasts (CFU-Fs) and results from these studies have been conflicting. Some reports showed that PTH injection has no effect on CFU-F number derived from bone marrow [9], [10], while others suggest that the hormone increases CFU-Fs, especially the number of alkaline phosphatase-positive CFU-F colonies [11], [12], [13]. A decrease in CFU-F number was also observed in mice after a single injection of PTH, which was explained by the PTH-induced adherence of mesenchymal progenitors to the bone surface [14].

Due to their self-renewal and differentiation abilities, mesenchymal progenitors hold great promise for tissue regeneration and gene therapy. Injected progenitors migrate specifically to sites of injury, inflammation, and tumor growth. Hence, the migratory behavior of mesenchymal progenitors has been extensively documented, but the signals guiding this migration and the pathways regulating it are still largely unknown. Mesenchymal stem cells may reside within a perivascular niche in the bone marrow (reviewed in [15], [16], [17]) and osteoprogenitors are in the bone marrow. Therefore, to become osteoblasts, they must migrate away from their bone marrow location toward the bone surface. Recent studies from Cao's group found that PTH stimulates the migration of these cells to the bone surface through the release of the chemotactic factor TGFβ1 from the bone matrix [18].

We hypothesize that PTH can also directly stimulate the release of chemotactic factors for mesenchymal progenitors from mature osteoblasts. Indeed, we demonstrate that PTH stimulates osteoblasts and osteocytes to release potent chemotactic factor(s) for mesenchymal progenitors in vitro. Using a number of approaches, including inhibitor, neutralizing antibody, and siRNA, we identified this chemotactic factor as amphiregulin, which acts on the epidermal growth factor receptor (EGFR) in mesenchymal progenitors to promote their migration in vitro. EGFR is a tyrosine kinase receptor that, upon ligand binding, activates several important intracellular signal transduction pathways such as Ras-Raf-MAP-kinase and PI-3-kinase-Akt and modulates a variety of cell functions such as proliferation, survival, adhesion, migration and differentiation [19]. Recent studies demonstrate that this signaling pathway plays an important role in bone metabolism (reviewed in [20]). Here, we demonstrate that, in vivo, the loss of EGFR activity specifically in osteoprogenitors/osteoblasts inhibited PTH's stimulatory action on bone formation. Overall, our findings suggest an important role of the EGFR signaling pathway in regulating the anabolic response to PTH.

## Materials and Methods

### Chemicals and Reagents

Rat PTH(1–34) was purchased from Bachem whereas human PTH(1–34) was synthesized by the MGH Peptide Core Facility. Recombinant human EGF, TGFα, HB-EGF, and amphiregulin were purchased from R&D Systems. SB202190, wortmannin, U0126, GM6001, and PD153035 were obtained from Calbiochem. Gefitinib was a product of LC Laboratories.

### Isolation and culture of human and rat bone marrow mesenchymal progenitors

Human bone marrow aspirates were purchased from Allcells and purified through Ficoll gradient (GE Healthcare) to obtain bone marrow mononuclear cells. These cells were seeded and grown in α minimal essential medium (αMEM) supplemented with 20% fetal bovine serum (FBS), 2 mM L-glutamine, and 100 µM L-ascorbate-2-phosphate for about 2 weeks to establish the adherent culture. To obtain rat mesenchymal progenitors, Sprague-Dawley rats at 1-month-old (Charles River) were euthanized by CO_2_ inhalation and the bilateral femora and tibiae were dissected under sterile conditions and washed in αMEM. Bone marrow cells were collected by flushing media through bones, filtered through a 70-µm cell strainer, and cultured in αMEM containing 10% FBS. Only passages less than five were used in the experiments. The human and rat mesenchymal progenitors had a fibroblast-like morphology in culture, were homogeneously CD90^+^CD105^+^CD146^+^CD45^−^ (human) and CD90^+^CD49e^+^CD45^−^CD34^−^ (rat) (data not shown). All animal work performed in this report was approved by the Institutional Animal Care and Use Committee (IACUC) at the University of Pennsylvania and Massachusetts General Hospital.

### Collection of conditioned media

UMR 106-01 cells were maintained in MEM with 5% FBS. Rat primary calvarial osteoblastic cells were obtained from neonatal calvariae by sequential digestions with collagenase and trypsin as described previously [21] and cultured in MEM containing 10% FBS until confluence. Then the media was switched to osteoblast differentiation media (BGJb medium containing 10% FBS, 10 mM β-glycerophosphate, and 50 µg/ml ascorbic acid) for 1 week. MC3T3-E1 cells were maintained in αMEM with 10% FBS. After confluence, cells were cultured in osteoblast differentiation media (αMEM with 10% FBS and 50 µg/ml ascorbic acid) for 4 days. All above cultures were incubated at 37°C in a humidified atmosphere containing 5% CO_2_. The osteocytic cells Ocy491 were isolated from long bones of 8 kb DMP1-GFP transgenic mice expressing green fluorescent protein (GFP) specifically in osteocytes and carrying an immortalizing antigen (tsAgSV40). Briefly, long bones (femora and tibiae) of 4-weeks old double transgenic (8 kb DMP1-GFP;tsAgSV40) mice were dissected under sterile condition and cells were isolated by sequential collagenase and EDTA digestions followed by FACS sorting for GFP-positive cells. GFP-positive sorted cells displayed the dendritic and stellate morphology characteristic of an osteocytic population. They were maintained in αMEM with 10% FBS at 33°C for proliferation and switched to 37°C to inactivate tsAg expression at confluence. After 2 weeks in culture at non permissive condition, Ocy491 cells express high levels of osteocytic markers SOST, E11 and DMP1 and a very low level of osteoblastic marker Kera compared to primary osteoblastic cells, as assessed by qRT-PCR (data not shown). According to this pattern of gene expression and, as previously described, Ocy491 can be classified as young osteocytes [22].

To collect conditioned media, cells were washed once with αMEM and then maintained in serum-free αMEM overnight followed by PTH treatment (10 nM human PTH(1–34) for Ocy491 cells and 10 nM rat PTH(1–34) for all other cells unless otherwise stated). Conditioned media were then harvested 4 hrs after addition of PTH and centrifuged at 3000 rpm for 10 min at 4°C to remove cell debris before storage at −80°C. Conditioned media collected from vehicle-treated cells were used as control.

### Chemotaxis assay

To measure the migration of total bone marrow cells, rat bone marrow cells flushed from long bones were washed with αMEM once and then plated (8×10^6^ total bone marrow cells/well) in the upper chamber of Costar 6-well transwell plates with 3-µm pore polycarbonate membranes (Corning, NY). Conditioned media were loaded in the bottom wells. After incubating plates at 37°C in humidified 5% CO_2_ for 24 hrs, the cells in the upper chamber were removed by washing with phosphate-buffered saline (PBS) followed by cotton scrubbing. The migrated cells were collected from the bottom part of the membrane by trypsinization and from medium in the lower wells and then counted using a hemocytometer.

The chemotactic activity of conditioned media for mesenchymal progenitors was evaluated using a 96-well microchemotaxis Boyden chamber (Neuro Probe). Mesenchymal progenitors were lifted by trypsin, washed once in αMEM and resuspended in αMEM. Cells (5000 cells/25 µl) were added to the upper wells and conditioned media harvested from osteoblastic and osteocytic cells were added to the lower wells. The contents of the upper and lower wells were separated by a filter with 8-µm pores. The chamber was then incubated at 37°C in humidified 5% CO_2_ for 5 hr. After incubation, cells on the upper side of the filter were mechanically removed using soft paper. The migrated cells on the lower side were fixed and stained with 0.3% crystal violet in methanol. Under a microscope, a total of five fields per filter were counted to calculate the average cell number per field. The chemotactic index is defined by setting the number of migrated cells toward control conditioned medium or αMEM as 1. To test the effects of individual signaling pathways on the migration, mesenchymal progenitors were mixed with inhibitor or neutralizing antibody first and then immediately loaded into the upper wells for the chemotaxis assay.

### cAMP assay

UMR 106-01, human and rat mesenchymal progenitors were seeded in 60-mm dishes and treated with 10 nM rat PTH(1–34) for 15 min. Cell lysates were then harvested to measure cAMP contents normalized against total protein amounts using a Parameter Cyclic AMP Assay kit (R&D Systems).

### Immunoblotting

Whole cell extracts of mesenchymal progenitors were separated by SDS-PAGE and immunoblotted as previously described [23] with antibodies raised against total EGFR (Santa Cruz # sc-03), the phosphorylated form of EGFR (Cell Signaling # 3777), total Akt (Cell Signaling #9272), the phosphorylated form of Akt (Cell Signaling # 4051), total p38MAPK (Cell Signaling # 9212), the phosphorylated form of p38MAPK (Cell Signaling # 4511), and β-actin (Santa Cruz # sc-47778). Secondary antibodies were purchased from Santa Cruz (goat anti-mouse # sc-2005 and goat anti-rabbit # sc-2004).

### Flow cytometry analysis

To detect the EGFR surface expression, ficoll gradient-purified human bone marrow cells or cultured mesenchymal progenitors were washed with PBS and resuspended in PBS containing 2% FBS. The cells were then incubated with PE-conjugated antibody for EGFR (BD Biosciences) for 45 min on ice. They were washed with PBS and analyzed by a flow cytometer (Beckman Coulter).

### Radiolabeled EGF ligand binding and calculation of EGFR number in mesenchymal progenitors

Human mesenchymal progenitors were seeded in 24-well plates overnight. On the next day, cells were washed twice with PBS and incubated with αMEM containing 1 mg/ml BSA and a series of concentrations of mouse ^125^I-EGF (GE Healthcare) for 2 hr at 37°C. After washing twice with PBS, cells were lysed in 0.5 M NaOH and the amount of radioactivity was counted in a gamma counter (1282 CompuGamma CS, LKB Wallac). Nonspecific binding was calculated as the radioactivity remaining bound to the cells in the presence of 250-fold cold EGF. Cell numbers were counted in a hemocytometer after they had been harvested from separate replicate plates. To calculate the number of EGFR on mesenchymal progenitors and the Kd value, a saturation curve was generated and analyzed using nonlinear regression (Graphpad, Prism5).

### siRNA knockdown of EGFR and amphiregulin

DsiRNA Tri*FECT*a Kits (IDT), including HSC.RNAI.N005228.12 for human EGFR and HSC.RNAI.N017123.12 for rat amphiregulin, were used to knockdown the EGFR expression in human mesenchymal progenitors and amphiregulin expression in UMR 106-01 cells, respectively, according to the manufacturer's protocol. Two to three days later, cells were harvested for RNA, protein lysates, or migration assay.

### RNA isolation and qRT-PCR

Total RNA was isolated from cells using Tri Reagent (Sigma-Aldrich). A Taqman® Reverse Transcription kit (Applied Biosystems) was used to reverse transcribe mRNA into cDNA. Following this, qPCR was performed using a Power SYBR® Green PCR Master Mix kit (Applied Biosystems). Primer sequences are listed in Supplemental Table S1.

### Skeletal characterization and PTH anabolic treatment in osteoprogenitor/osteoblast-specific EGFR deficient mice

Col-Cre *Egfr^Wa5/flox^* mice and their controls were generated as described previously [24]. Briefly, we bred Col 3.6-Cre with *Egfr^Wa5/+^* to obtain Col-Cre *Egfr^Wa5/+^*. These mice were then crossed with *Egfr^flox/flox^* to generate Col-Cre *Egfr^Wa5/flox^* mice and their Wa5 (*Egfr^Wa5/flox^*) and wild-type (Col-Cre *Egfr^flox/+^* and *Egfr^flox/+^*) littermates. Four-month-old mice were injected daily with either vehicle (saline) or PTH (100 µg/kg/day) for 5 weeks (5 days per week). There was no significant difference in body weight among genotype groups and between treatments. One day after the last injection, both tibiae were harvested, one for peripheral quantitative computed tomography (pQCT) analysis using an XCT Research SA (Stratec Medizintechnik, Pforzheim, Germany) followed by micro-computed tomography (microCT) measurement using Skyscan 1172 high resolution microCT (Skyscan, Belgium), and another one for bone histomorphometry. The experimental details were described previously [24].

### Statistical analysis

All results are expressed as means ± SEM of triplicate measurements with all experiments being repeated independently at least three times. Unpaired Student's t test was used to evaluate the statistical difference between two groups. In cases of multiple groups, differences were analyzed with one-way analysis of variance (ANOVA) with Bonferroni post test. Values of p<0.05 were considered significant. Statistical analysis was performed with GraphPad Prism5.

## Results

### Bone marrow mesenchymal progenitors migrate toward PTH-stimulated osteoblasts and osteocytes

**Figure 1:**
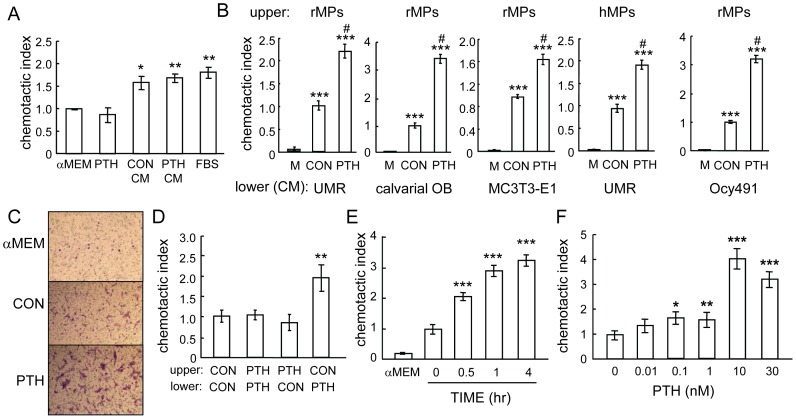
Conditioned media from PTH-treated osteoblastic cells contain chemotactic factors for bone marrow mesenchymal progenitors. (A) Migration of freshly flushed rat bone marrow cells toward PTH and conditioned media from UMR 106-01 cells. Bone marrow cells flushed from rat long bones were immediately seeded in the upper chamber of transwell plates. The bottom wells were loaded with media alone (αMEM), media containing 10 nM PTH (PTH), or conditioned media (CM) collected from UMR106-01 cells that had been treated with control (CON) or 10 nM PTH (PTH) for 4 hr. The number of cells that migrated to the bottom wells was counted 24 hr later. αMEM containing 5% FBS (FBS) was used as a positive control for cell migration. *: p<0.05; **: p<0.01 vs. αMEM. (B) Conditioned media from various PTH-treated osteoblastic and osteocytic cells stimulated the migration of either human or rat mesenchymal progenitors (MP) in the Boyden chamber assay. The cells seeded in the upper wells are depicted at the top and conditioned media loaded in the lower wells are shown at the bottom. M: αMEM. ***: p<0.001 vs. αMEM; &: p<0.01; #: p<0.001 vs. CON. (C) Microscopic images of the mesenchymal progenitors that migrated to the lower sides of filters in the assay depicted in B. (D) The migration of mesenchymal progenitors stimulated by conditioned media from PTH-treated osteoblasts is not chemokinesis. Mesenchymal progenitors were suspended in conditioned media harvested from either control or PTH-treated UMR 106-01 cells and seeded in the upper chambers. The lower wells were filled with conditioned media, resulting in a total of 4 combination types. **: p<0.01 vs. CON/CON. (E) Time course of the release of chemotactic factor(s) from osteoblasts after PTH treatment. UMR 106-01 cells were treated with PTH and conditioned media were harvested at indicated time points and loaded in the lower wells for chemotaxis assays. ***: p<0.001 vs. time 0 hr. (F) The dosage effects of PTH on chemotactic factor(s) release from osteoblasts. UMR 106-01 cells were treated with different doses of PTH for 4 hr and then conditioned media were harvested for chemotaxis assays. *: p<0.05; **: p = 0.06; ***: p<0.001 vs. 0 nM.

We initially performed migration assays using freshly harvested rat bone marrow cells. We found that while a significant number of bone marrow cells (26.7% of the cells plated in the upper chamber) migrated toward αMEM, there were more cells (41.4%) that migrated toward conditioned media collected from UMR 106-01, a rat osteoblastic cell line (Fig. 1A). However, we did not observe any further increase in the number of migrated cells when conditioned media from PTH-treated UMR 106-01 cells were tested (Fig. 1A), suggesting that osteoblastic culture media contain migration factor(s) for bone marrow cells.

Since bone marrow mesenchymal progenitors are a rare subpopulation of the total bone marrow with an approximate number of one in 10^5^–10^6^ bone marrow mononuclear cells [25], we could not conclude based on the total bone marrow migration results whether the conditioned media promoted the migration of mesenchymal progenitors. To specifically observe the migration of mesenchymal progenitors, we expanded these cells in culture and then used them for the migration assay. As shown in Fig. 1B (left panel) and C, very few rat mesenchymal progenitors migrated toward αMEM alone whereas conditioned media from UMR 106-01 cultures contained factor(s) that stimulated their migration across the filter. Interestingly, conditioned media from PTH-treated UMR 106-01 cells resulted in more than a 2-fold increase in the number of migrated mesenchymal progenitors, suggesting that PTH treatment increases the amount of migratory factor(s) for mesenchymal progenitors in the conditioned media.

To confirm that this response is not limited to UMR 106-01 cells but is a general response of osteoblastic cells to PTH, we tested two other osteoblastic cells: rat primary calvarial osteoblasts and murine osteoblastic cells, MC3T3-E1. These cells start as osteoprogenitors and have the ability to undergo osteoblastic differentiation in vitro. We collected conditioned media at the differentiation stage with or without PTH treatment and used them for the chemotaxis assay. Consistent with the above UMR 106-01 cell results, conditioned media from PTH-treated primary osteoblasts and MC3T3-E1 cells led to a 3.2- and 1.6-fold increase, respectively, in mesenchymal progenitor migration compared to non-treated conditioned media (Fig. 1B). A similar effect was observed with human mesenchymal progenitors (Fig. 1B). Osteocytes are terminally differentiated osteoblasts trapped in the bone matrix. They form an extensive connecting network through which they are capable of controlling osteoblast and osteoclast formation at the bone surface. Interestingly, we found that conditioned media from PTH-treated osteocytic Ocy491 cells also promoted the migration of mesenchymal progenitors (2.8-fold, Fig. 1B right panel), suggesting that PTH not only acts on mature osteoblasts but also on osteocytes to stimulate the release of migratory factor(s) for mesenchymal progenitors. In the following experiments, unless otherwise stated, similar results between rat and human mesenchymal progenitors were observed.

To distinguish whether this phenomenon is chemotaxis (concentration-dependent cell migration) or chemokinesis (random cell motility), we suspended mesenchymal progenitors in conditioned media from either control or PTH-treated UMR 106-01 cells and plated them in the upper wells. The lower wells were filled with conditioned media from either control or PTH-treated UMR 106-01 cells. Among all four possible combinations, the number of migrated cells in the chambers with control conditioned media in the upper well and PTH-treated conditioned media in the lower well was the highest (Fig. 1D). This result indicates that PTH-induced migratory factor(s) in osteoblast culture media are chemoattractant(s) for mesenchymal progenitors.

Next, we examined the time course of the chemotactic factor release from PTH-treated cells. As shown in Fig. 1E, the release of chemoattractants seems to be a very rapid process since conditioned media from cells treated with PTH as early as 30 min contained a high enough amount of factor(s) to significantly increase the migration of mesenchymal progenitors. The chemotactic activity of conditioned media continuously increased up to 4 hr after PTH treatment. Dose response experiments revealed that conditioned media from cells treated with as little as 0.1 nM PTH was able to significantly stimulate the chemotaxis of mesenchymal progenitors, but the peak occurred around 10 nM PTH (Fig. 1F). In summary, our data clearly demonstrate that PTH stimulates osteoblastic and osteocytic cells to produce soluble chemoattractants for bone marrow mesenchymal progenitors.

### PTH itself is not a chemotactic factor for mesenchymal progenitors

**Figure 2:**
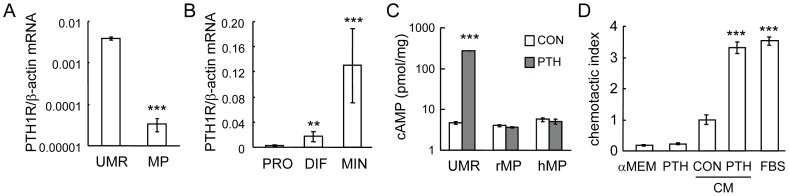
PTH itself is not a chemotactic factor for mesenchymal progenitors. (A) qRT-PCR quantification of mRNA levels of PTH1R in UMR106-01 cells and rat mesenchymal progenitors in culture. ***: p<0.001 vs. UMR. (B) The expression of PTH1R in rat calvarial osteoblasts increases dramatically during their osteogenic differentiation as measured by qRT-PCR. PRO: proliferation stage; DIF: differentiation stage; MIN: mineralization stage. **: p<0.01; ***: p<0.001 vs. PRO. (C) PTH stimulates cAMP production in UMR 106-01 cells but not mesenchymal progenitors. ***: p<0.001 vs. con. (D) PTH alone does not stimulate the migration of mesenchymal progenitors. Chemotaxis assays were performed with lower wells filled with αMEM, αMEM containing 10 nM PTH, conditioned media from control- and PTH-treated UMR 106-01 cells, and αMEM containing 5% FBS. ***: p<0.001 vs. CON.

Since we did not remove PTH from the conditioned media before the migration assay, it is possible that PTH might be a direct chemotactic factor for mesenchymal progenitors. To exclude this possibility, we first examined the PTH1R expression in mesenchymal progenitors. Compared to osteoblastic UMR 106-01 cells, rat mesenchymal progenitors only express 0.62% of PTH1R mRNA as shown by qRT-PCR (Fig. 2A). In culture, rat primary calvarial osteoblastic cells go through three stages: proliferation (the first week), differentiation (the second week), and mineralization (the third week). qRT-PCR demonstrated that PTH1R expression was strongly elevated during this osteoblastic differentiation process with more than a 34-fold increase at the mineralization phase compared to the proliferation phase (Fig. 2B). These results clearly indicate that PTH1R is expressed at a much higher level in mature osteoblasts compared to mesenchymal progenitors, which is consistent with previous reports [26], [27]. The major signaling pathway activated by PTH in osteoblasts is the cAMP/PKA pathway. While it led to a 58-fold increase in cAMP in UMR 106-01 cells, PTH treatment did not increase cAMP production in mesenchymal progenitors (Fig. 2C), indicating that mesenchymal progenitors do not have a detectable response to PTH stimulation. Furthermore, we performed migration assays adding only PTH to the bottom wells and found that it has no chemotactic activity toward mesenchymal progenitors (Fig. 2D). Meanwhile, there was a 3-fold increase in the number of cells that migrated toward conditioned media from PTH-treated samples compared to that toward control conditioned media, and this number was comparable to that toward serum-containing medium (Fig. 2D). Moreover, PTH itself did not enhance the migration of total bone marrow cells (Fig. 1A). These data clearly indicate that it is a PTH-induced downstream target, and not PTH itself, that stimulates the chemotaxis of mesenchymal progenitors.

### Blocking PI3K and p38MAPK activities in mesenchymal progenitors abolishes their migration toward PTH-treated osteoblasts

**Figure 3:**
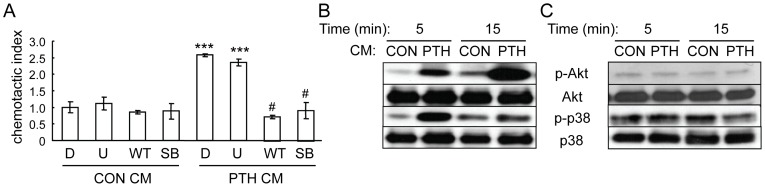
PI3K/Akt and p38MAPK pathways are required for the migration of mesenchymal progenitors toward conditioned media from PTH-treated osteoblastic cells. (A) Chemotaxis assays were performed with mesenchymal progenitors and conditioned media from either control- or PTH-treated UMR 106-01 cells in the presence or absence of pathway-specific inhibitors. D: DMSO; U: U0126 (20 µM); WT: wortmannin (3 µM); SB: SB202190 (20 µM). Inhibitors were added to both upper and bottom chambers. ***: p<0.001 vs. CON CM D; #: p<0.001 vs. PTH CM D. (B) Conditioned media from PTH-treated UMR 106-01 cells stimulated the phosphorylation of Akt and p38MAPK in MSCs. (C) PTH alone did not activate Akt and p38MAPK pathways in mesenchymal progenitors.

To identify the chemotactic factors released from PTH-stimulated osteoblasts, we first used inhibitors to study which intracellular signaling pathways in mesenchymal progenitors are important for mediating their migration toward PTH-stimulated osteoblasts. As shown in Fig. 3A, while inhibitors for MAPK (U0126), PI3K (wortmannin), and p38MAPK (SB202190) had no effect on the migration of mesenchymal progenitors toward normal osteoblastic conditioned media, wortmannin and SB202190, but not U0126, completely blocked the increase in mesenchymal progenitor migration toward conditioned media from PTH-treated osteoblasts, suggesting that PI3K/Akt and p38MAPK pathways are both essential for the action of PTH-released chemotactic factors from osteoblasts. Furthermore, we collected the conditioned media from osteoblasts and added them to the mesenchymal progenitor culture. Western blots showed rapid and strong increases in both phosphorylated Akt and p38MAPK amounts in mesenchymal progenitors after being treated with conditioned media from PTH-treated osteoblastic cells (Fig. 3B). However, PTH itself had no detectable effect on these pathways within 15 min of treatment (Fig. 3C), providing additional evidence that PTH does not directly act on mesenchymal progenitors to promote their migration.

### EGFR signaling in mesenchymal progenitors is critical for their migration toward PTH-treated osteoblasts

We have previously shown that amphiregulin is an immediate-early gene induced by PTH in osteoblasts [21]. Amphiregulin belongs to a family of epidermal growth factor (EGF)-like ligands which also includes EGF, transforming growth factor α (TGFα), betacellulin, heparin-binding-EGF (HB-EGF) and epiregulin. Since the receptor for these ligands, EGFR, plays a critical role in cancer cell migration and tumor metastasis [28], we investigated whether EGFR signaling in the mesenchymal progenitors mediates the chemotactic effects of conditioned media from PTH-treated osteoblasts.

**Figure 4:**
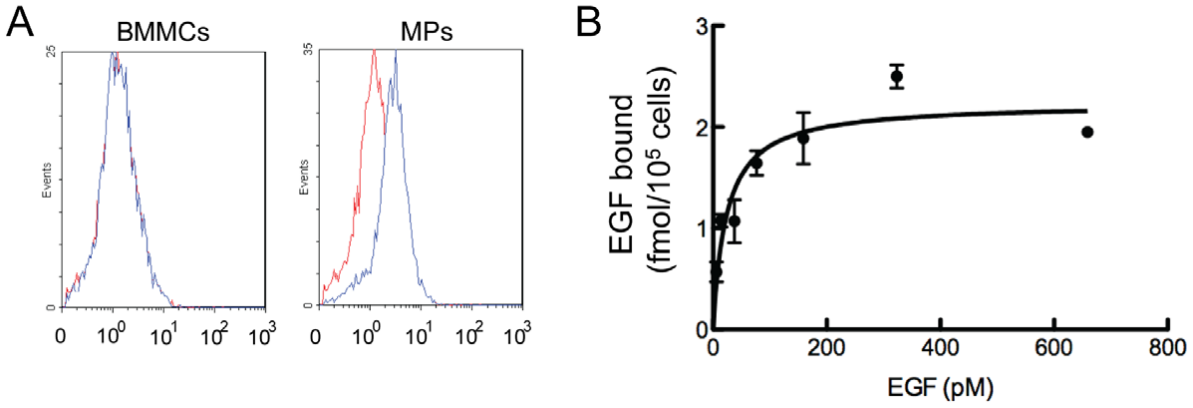
Mesenchymal progenitors express EGFR. (A) Flow cytometry analyses demonstrate that cultured mesenchymal progenitors (MPs) but not freshly isolated bone marrow mononuclear cells (BMMCs) express EGFR surface antigen. Blue curve: EGFR antibody; red curve: isotype control. (B) Saturation curve of binding of ^125^I-EGF to mesenchymal progenitors.

Although the EGFR is critical for the mobilization of hematopoietic stem cells [29], we found no detectable amount of cell surface EGFR on freshly flushed bone marrow cells (Fig. 4A), suggesting that the majority of hematopoietic lineage cells lack the expression of EGFR. However, after culture, the plastic adherent bone marrow mesenchymal progenitors are EGFR-positive cells (Fig. 4A), which is consistent with previous reports that EGF-like ligands regulate mesenchymal progenitor proliferation, differentiation, and apoptosis [30], [31]. The quantitative expression level of EGFR in mesenchymal progenitors has not been studied. We performed receptor-ligand binding assays using ^125^I-labeled EGF and subsequent nonlinear regression analyses which revealed a single class of EGF binding sites on mesenchymal progenitors with a dissociation constant of 34.9 pM and receptor number of 1.4×10^4^ per cell (Fig. 4B).

**Figure 5:**
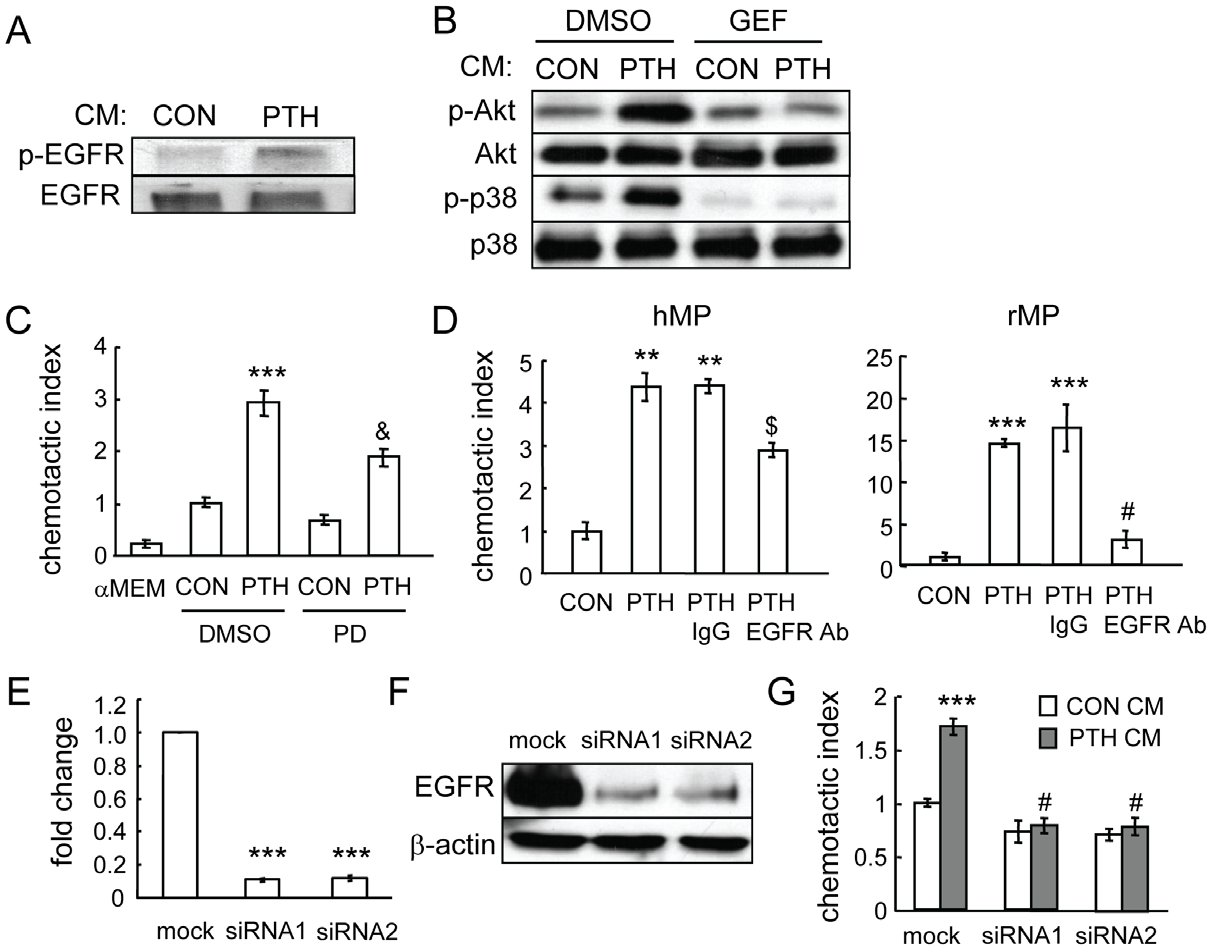
EGFR signaling mediates the chemotactic effect of PTH on mesenchymal progenitors. (A) Conditioned media from PTH-treated UMR 106-01 cells increased the phosphorylation of EGFR in mesenchymal progenitors. In this experiment, mesenchymal progenitors were treated with conditioned media for 5 min and then lysed for Western blot. (B) The enhanced phosphorylation of Akt and p38MAPK in mesenchymal progenitors by conditioned media from PTH-treated UMR106-01 cells is dependent on the EGFR pathway. Mesenchymal progenitors were pre-incubated with either DMSO or gefitinib (10 µM, GEF) for 30 min followed by addition of conditioned media to the culture. Cell lysates were collected 5 min later for Western blot analyses. (C) The EGFR inhibitor PD153035 (10 µM, PD) was added to both the upper and lower wells of the chemotaxis assay and partially blocked the PTH-induced chemotactic activity of conditioned media from UMR 106-01 cells. ***: p<0.001 vs. DMSO CON; &: p<0.01 vs. DMSO PTH. (D) An EGFR neutralizing antibody (4 µg/ml) was mixed with mesenchymal progenitors before the chemotaxis assay and suppressed the migration of mesenchymal progenitors towards conditioned media from PTH-treated UMR 106-01 cells. IgG: isotype control. **: p<0.01; ***: p<0.001 vs. CON; $: p<0.05; #: p<0.001 vs. PTH. (E) qRT-PCR demonstrates the knockdown of EGFR mRNA levels in mesenchymal progenitors by siRNAs. ***: p<0.001 vs. MOCK. (F) Immunoblotting reveals that the EGFR protein level was dramatically decreased in mesenchymal progenitors transfected with siRNAs for EGFR. (G) Blocking of EGFR expression in mesenchymal progenitors by siRNAs abolished the chemotactic migration of these cells toward conditioned media from PTH-treated UMR 106-01 cells. ***: p<0.001 vs. mock CON; #: p<0.001 vs. mock PTH.

To determine if the activation of EGFR is important for PTH-induced mesenchymal progenitor migration, cells were treated with osteoblast conditioned media and EGFR phosphorylation was analyzed. Mesenchymal progenitors treated with conditioned media from PTH-treated osteoblasts had increased phosphorylated EGFR compared to control (Fig. 5A). Addition of an EGFR-specific inhibitor, gefitinib, abolished the enhanced levels of phosphorylated Akt and p38MAPK (Fig. 5B), suggesting that activation of EGFR in mesenchymal progenitors is crucial for promoting both PI3K/Akt and p38MAPK pathways. Moreover, addition of the EGFR inhibitor PD153035 to both the upper and lower wells of the Boyden chamber partially blocked the chemotactic activity of PTH-treated conditioned media (Fig. 5C). PD153035 alone had no effect on the cell migration toward control conditioned media. Similarly, when they were pretreated with an EGFR neutralizing antibody, the mesenchymal progenitors had decreased migration toward PTH-treated conditioned media with a larger decrease observed by rat mesenchymal progenitors than human mesenchymal progenitors (Fig. 5D). Control IgG had little effect on this migration. Lastly, we used a siRNA approach to decrease the expression of EGFR in mesenchymal progenitors. Two siRNAs against different regions of the human EGFR coding region were transfected separately into mesenchymal progenitors. qRT-PCR and Western blots showed that both of them efficiently knocked down the EGFR expression at both mRNA and protein levels (more than 90% decrease) (Fig. 5E and F). Subsequent chemotaxis assays revealed that, while mock siRNA-transfected mesenchymal progenitors still migrated more toward PTH-treated conditioned media, EGFR-specific siRNA-transfected mesenchymal progenitors no longer exhibited preference toward this media (Fig. 5G). Taken together, our data clearly show that EGFR activity in mesenchymal progenitors at least partially mediates their migration toward PTH-treated osteoblasts.

### Amphiregulin released from PTH-treated osteoblastic cells recruit mesenchymal progenitors

**Figure 6:**
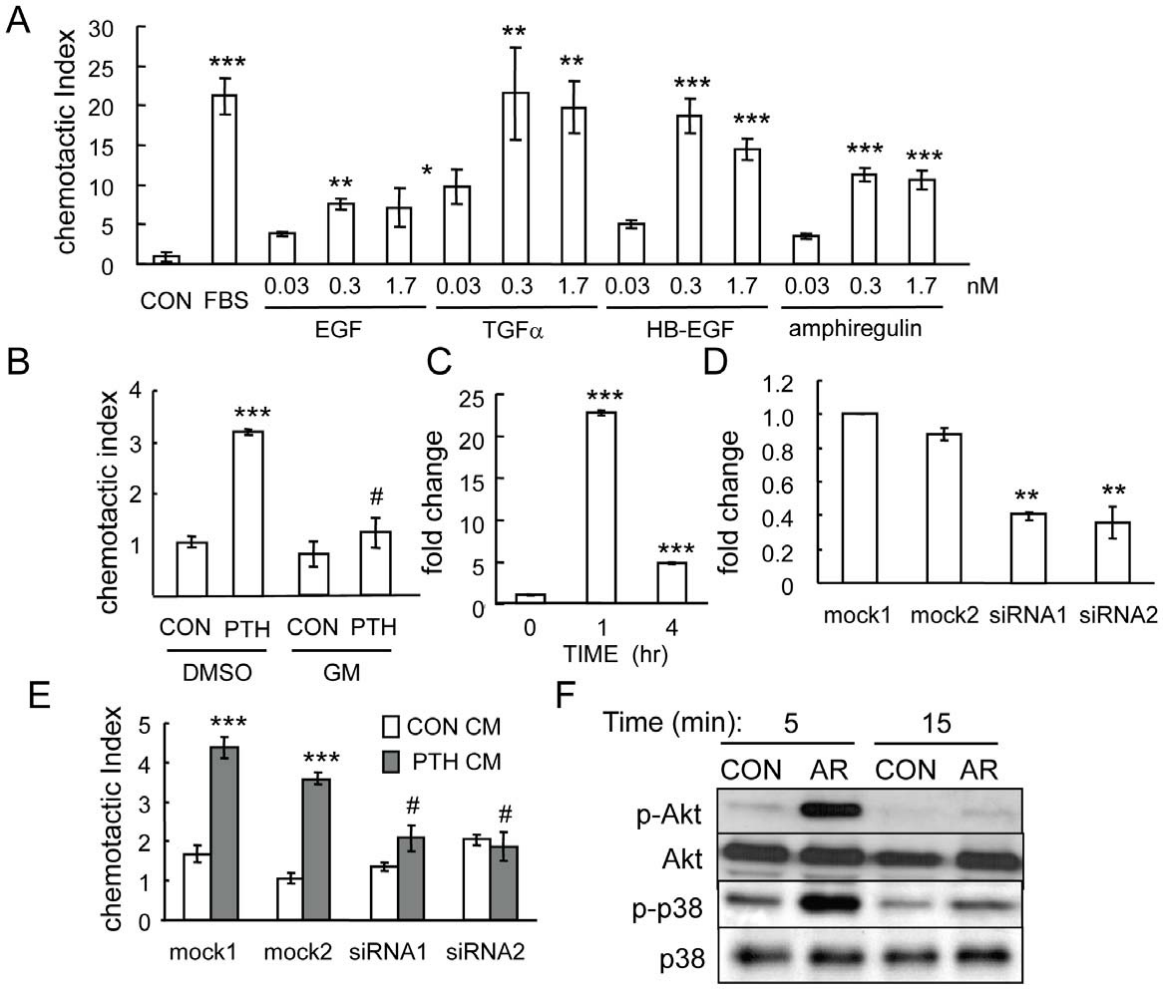
PTH stimulates the release of amphiregulin from osteoblasts to promote mesenchymal progenitor migration. (A) EGF-like ligands are chemotactic factors for mesenchymal progenitors. αMEM containing various amounts of EGF-like ligands was added in the lower wells of chemotaxis assays using rat mesenchymal progenitors. αMEM containing 5% FBS was used as positive control. *: p<0.05; **: p<0.01; ***: p<0.001 vs. CON. (B) Addition of GM6001 (10 µM, GM) in the chemotaxis assay blocked the migration of mesenchymal progenitors toward conditioned media from PTH-treated UMR 106-01 cells. ***: p<0.001 vs. CON DMSO; #: p<0.001 vs. PTH DMSO. (C) qRT-PCR shows that PTH (10 nM) induced the expression of amphiregulin in osteocytic Ocy491 cells at 1 h. (D) qRT-PCR demonstrates the knockdown of amphiregulin mRNA in UMR 106-01 cells after 1 hr of PTH (10 nM) treatment by siRNAs. **: p<0.01 vs. mock1. (E) Chemotaxis assays reveal that PTH did not stimulate the release of chemotactic factor(s) from UMR106-01 cells transfected with siRNAs for amphiregulin. ***: p<0.001 vs. CON CM; #; p<0.001 vs PTH CM mock1. (F) Amphiregulin (AR) stimulated Akt and p38MAPK phosphorylation in mesenchymal progenitors as shown by immunoblotting.

To identify the ligands responsible for activation of EGFR in mesenchymal progenitors, we performed chemotaxis assays with four major EGF-like ligands (EGF, HB-EGF, TGFα and amphiregulin) and found that all of them are potent chemotactic factors for mesenchymal progenitors (Fig. 6A). Among them, HB-EGF and TGFα have the strongest effects since the number of cells that migrated toward 0.3 nM of these ligands was comparable to that toward serum. EGF exhibited the least potent effect, but the number of cells that migrated toward 0.3 nM EGF was still 7.6-fold more than that toward media alone.

GM6001 is a general inhibitor for metalloproteinases including MMP (matrix metalloproteinase), adam (a distintegrin and metalloproteinase), and adamts (a disintegrin and metalloproteinase with thrombospondin motif), which play important roles in ectodomain shedding of membrane-anchored growth factors, including all EGF-like ligands [32], [33]. Interestingly, we found that pretreatment of UMR 106-01 cells with GM6001 also impaired the chemotactic activity of PTH-treated conditioned media (Fig. 6B), implying that PTH-released osteoblastic chemotactic factor(s) might originally exist in a membrane-bound form.

Our previous studies reveal that among all EGF-like ligands, amphiregulin is the most up-regulated by PTH in osteoblasts [21]. Since the above mentioned migration assays (Fig. 1B) found that PTH-treated osteocytes secreted more chemotactic factors for mesenchymal progenitors compared to vehicle-treated cells, we next examined whether PTH stimulates amphiregulin expression in these cells. Indeed, qRT-PCR validated that amphiregulin mRNA was highly stimulated by PTH in Ocy491 cells (29-fold) at 1 hr (Fig. 6C). To investigate the role of amphiregulin in this PTH-induced mesenchymal progenitor migration, knockdown of amphiregulin was performed by transfecting UMR 106-01 cells with two different non-specific siRNAs or two siRNAs against different regions of the amphiregulin coding sequence. qRT-PCR revealed that amphiregulin-specific siRNAs inhibited PTH-induced amphiregulin expression more than 60% (Fig. 6D). Interestingly, conditioned media collected from these cells pre-treated with PTH lost their chemotactic activity for mesenchymal progenitors (Fig. 6E), suggesting that amphiregulin from osteoblastic cells and EGFR from mesenchymal progenitors constitute the major signaling pathway mediating the PTH-induced migration of mesenchymal progenitors toward osteoblasts and osteocytes. Consistent with these results, we also found that amphiregulin is able to strongly activate the PI3K/Akt and p38MAPK pathways in mesenchymal progenitors (Fig. 6F).

### Loss of EGFR activity in osteoprogenitors/osteoblasts attenuates the anabolic response to PTH

**Figure 7:**
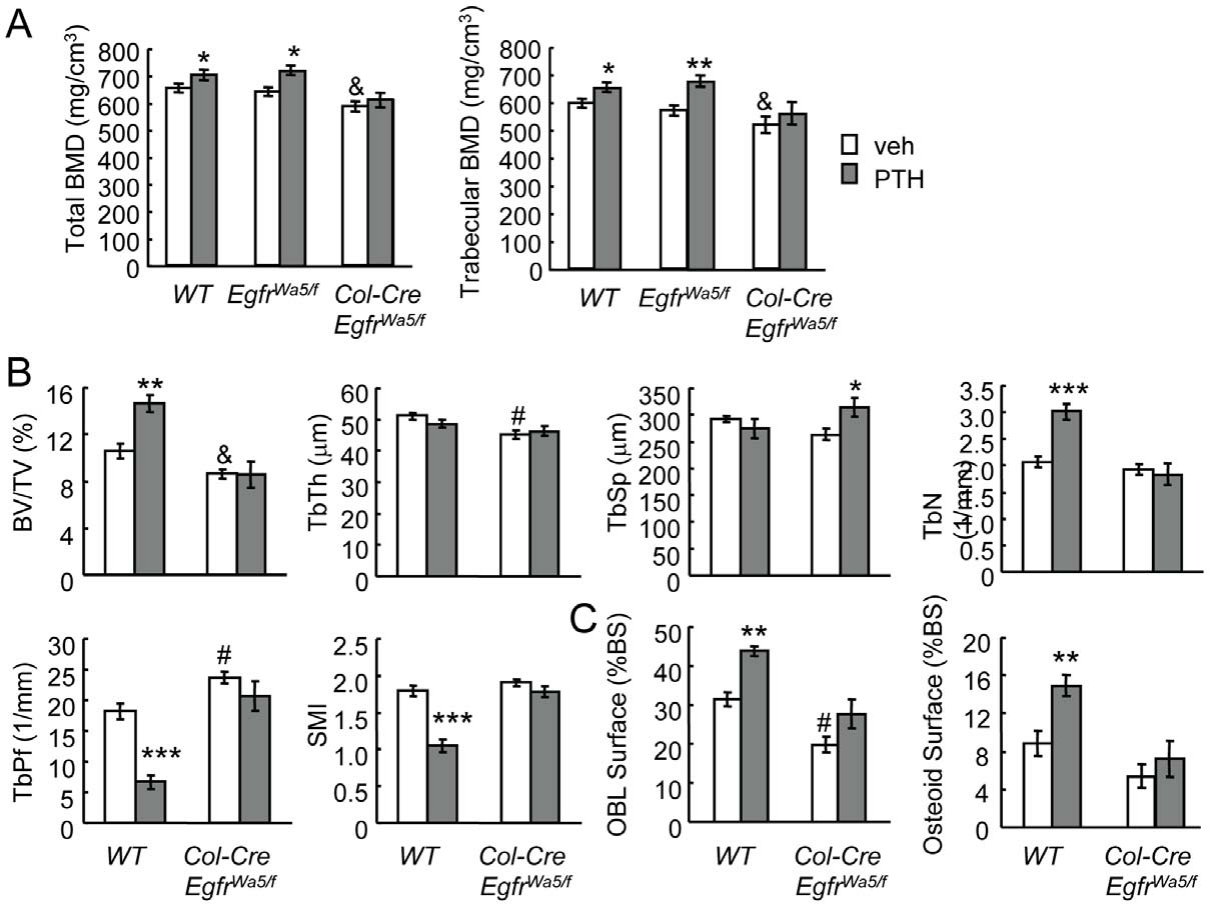
The anabolic actions of PTH on trabecular bone are attenuated in EGFR-deficient mice. (A) pQCT measurement of total and trabecular BMDs of the proximal tibiae of vehicle- or PTH-injected *Col-Cre Egfr^Wa5/flox^* mice, their Wa5 (*Egfr^Wa5/flox^*) and wild type (WT) siblings. (B) Structural parameters of trabecular bone in the proximal tibiae of vehicle- or PTH-injected *Col-Cre Egfr^Wa5/flox^* and WT mice. BV/TV: trabecular bone volume/tissue volume; TbTh: trabecular thickness; TbSp: trabecular separation; TbN: trabecular number; TbPf: trabecular pattern factor; SMI: structure model index. (C) Bone histomorphometry revealed that PTH-induced osteoblast formation is blunted in *Col-Cre Egfr^Wa5/flox^* mice. *: p<0.05; **: p<0.01; ***: p<0.001 PTH vs. veh; &: p<0.05; #: p<0.01 vehicle-treated *Col-Cre Egfr^Wa5/flox^* vs vehicle-treated WT mice. n = 5–7 mice per group.

The 3.6 kb alpha1(1) collagen promoter is activated early during osteogenic differentiation and expressed in osteoprogenitors [34]. As a result, Cre driven by this promoter targets osteoprogenitors and osteoblasts [35] and has been used successfully to knock down preosteoblast/osteoblast expression in a number of mouse models. Wa5 has a single missense mutation Asp833Gly in the highly conserved DFG domain of the EGFR kinase catalytic loop and codes for a kinase dead, dominant negative receptor [36]. While Wa5 homozygous mice, similar to *Egfr* null mice, are embryonic lethal, the heterozygous mice (*Egfr^Wa5/+^*) have significantly attenuated EGFR activity compared to wild-type mice [36] but the remaining low activity of EGFR is sufficient to maintain their viability and retain normal bone [24], [37]. We have previously demonstrated that, while the skeletal phenotype of *Col-Cre Egfr^flox/flox^* mice are indistinguishable from wild-type mice due to the incomplete conversion of floxed alleles by Cre, further inactivation of EGFR signaling by introducing the Wa5 allele (*Col-Cre Egfr^Wa5/flox^* mice) led to decreased EGFR activity in osteoprogenitors and reduced total and trabecular bone mineral density (BMD) at 3 and 7 months of age [24]. To determine if the reduction of EGFR activity in osteoprogenitors reduces the anabolic response to PTH, we treated *Col-Cre Egfr^Wa5/flox^* mice and their Wa5 and wide-type siblings with daily PTH injections for 5 weeks. pQCT analyses of proximal tibiae showed that PTH significantly increased total and trabecular BMDs in wild-type and Wa5 mice but had no effects on BMDs of *Col-Cre Egfr^Wa5/flox^* mice (Fig. 7A). This finding is consistent with a previous report that *Egfr^Wa5/+^* mice respond to PTH anabolic injections normally [38] and therefore we excluded *Egfr^Wa5/flox^* mice from further analyses. MicroCT measurements confirmed that PTH significantly increased bone volume/tissue volume fraction (BV/TV, 38.0%) and trabecular number (TbN, 45.8%) in the tibial trabecular bone and improved the structural integrity and mechanical strength of the trabecular bone by decreasing the trabecular pattern factor (TbPf, 63.5%) and surface model index (SMI, 42.8%) in wild-type mice but had no significant effects on these parameters in the *Col-Cre Egfr^Wa5/flox^* mice (Fig. 7B). Interestingly, bone histomorphometry revealed that PTH increased osteoblast surface (40.5%) and osteoid surface (53.6%) in wild-type mice but these effects were blunted in *Col-Cre Egfr^Wa5/flox^* mice (Fig. 7C), suggesting that PTH-induced osteoblast formation is suppressed by the loss of EGFR in osteoprogenitors. Consistent with our previous data [24], *Col-Cre Egfr^Wa5/flox^* mice had significantly decreased total and trabecular BMDs, BV/TV, TbTh and osteoblast surface compared to wide-type (Fig. 7).

## Discussion

**Figure 8:**
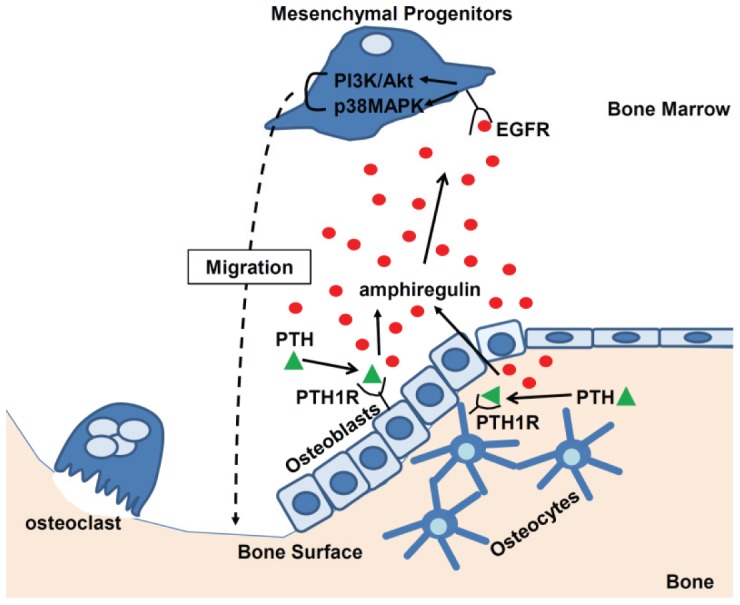
A model for PTH-induced mesenchymal progenitor migration in bone.

It is well established that intermittent injections of PTH stimulates both bone resorption and bone formation and leads to a net increase in bone formation. However, its exact mechanism of action, in particular its regulation of mesenchymal progenitors, is not completely understood. We tested mesenchymal progenitors from two sources (human and rat), osteoblasts from three sources (UMR 106-01, MC3T3-E1, and primary osteoblasts), and an osteocytic cell line (Ocy491), and found that PTH can act directly on osteoblastic and osteocytic cells to rapidly stimulate the release of chemotactic factors for mesenchymal progenitors in vitro. We also demonstrated that the reduction of EGFR activity in osteoprogenitors/osteoblasts in mice led to the inhibition of the bone anabolic response to PTH. As summarized in Fig. 8, we propose that PTH injection rapidly stimulates osteoblasts on the bone surface and osteocytes inside the bone matrix to produce the chemotactic EGF-like ligand, amphiregulin, which binds to the EGFR on mesenchymal progenitors in the bone marrow, promotes the activation of the PI3K/Akt and p38MAPK signaling pathways in those cells, and activates their migration toward the bone surface. We believe that this novel mechanism contributes to part of PTH's anabolic actions since multiple other mechanisms have been previously proposed [5], [8]. Our previous reports demonstrated that amphiregulin is a potent mitogen for mesenchymal progenitors [21] and that it inhibits their differentiation into mature osteoblasts [23], and therefore maintains these progenitors in an undifferentiated state. Once at the bone surface, these progenitors will then differentiate into mature osteoblasts under the influence of growth factors highly concentrated in the bone matrix, such as BMPs and IGFs, and ultimately contribute to the new bone formation associated with intermittent PTH injections.

Normal adult bone does not have modeling-based bone formation. However, bone formation with PTH appears to result from an increase in the rate of both remodeling and modeling (reviewed in [39], [40]). The mechanism proposed above could contribute to both types of bone formation. A recent finding from Wu et al. suggests a similar effect of PTH on mesenchymal progenitor migration but through a distinct mechanism [18]. They observed an increase in the migration of Sca-1-positive skeletal stem cells toward the bone surface after 1 week of PTH injections, which is dependent on PTH-induced bone resorption and subsequent TGFβ1 release from the bone matrix. Since our data show that PTH directly stimulates chemotactic factor secretion from osteoblasts and osteocytes, it would be interesting to examine whether amphiregulin and TGFβ1 are synergistic in recruiting mesenchymal progenitors toward bone surfaces. In addition, the notion that PTH stimulates the migration of mesenchymal progenitors toward the bone surface is also consistent with a new finding that PTH treatment relocates the small blood vessels in the bone marrow, which harbors the niche for MSCs, closer to the bone forming surface [41].

Previous reports and our data demonstrate that osteoblasts and osteocytes are the major target of PTH. In bone, PTH1R primarily exists in osteoblasts, osteocytes, and chondrocytes but not in osteoclasts [3], [4]. In situ hybridization revealed that the most intense areas for PTH1R transcript expression were a specific maturation stage of the growth plate, mature osteoblasts lining trabecular bones, and cortical periosteal and endosteal surfaces [4], [42]. More importantly, the expression of the PTH early-response gene c-fos is first strongly elevated in trabecular, periosteal and endosteal osteoblasts and chondrocytes within 15–30 min after one PTH injection and then followed by stromal cells and osteoclasts after 1–2 hr [42]. Our qRT-PCR data analyzing the expression of PTH1R in three different differentiation stages of rat calvarial primary osteoblastic cultures further demonstrate that the highest PTH1R expression occurs at the late osteoblast mineralization stage (Fig. 2B). This is also consistent with our previous observation that PTH-regulated genes, such as amphiregulin, usually exhibit much higher PTH-responsiveness in differentiated osteoblasts than in preosteoblasts [21]. The expression of PTH1R in osteocytes was first suggested by in situ hybridization [4] and its important role in regulating bone structures was later confirmed by either osteocyte-specific deletion [43] or targeted overexpression mouse models [44]. In addition, PTH-induced suppression of sclerostin in osteocytes is considered as one of the mechanisms by which PTH stimulates bone formation [45]. On the contrary, mesenchymal progenitors do express PTH1R but the level of PTH1R is so low that there was no detectable change of cAMP production in these cells after PTH stimulation. However, this conclusion does not exclude the possibility that PTH regulates mesenchymal progenitors directly in a migration-independent manner. It should be pointed out that untreated osteoblastic and osteocytic cells also secrete chemotactic factors for mesenchymal progenitors (Fig. 1B, comparing control and αMEM), suggesting that osteoblasts and osteocytes may play an important role in progenitor migration for normal bone remodeling independent of PTH treatment.

Our data strongly suggest that activation of EGFR by EGF-like ligands, especially amphiregulin, is vital for the migratory response of mesenchymal progenitors to PTH. Amphiregulin is one of the most upregulated genes after PTH treatment of osteoblasts. EGF and HB-EGF have been studied as exogenous growth factors for ex vivo expansion of the mesenchymal progenitor population [30], [31]. We previously have demonstrated that the EGFR signaling pathway is important for mesenchymal progenitor maintenance [24]. Here, we demonstrate that, unlike the majority of the cells that compose the bone marrow, the hematopoietic cells, mesenchymal progenitors express EGFR and are the main targets of EGF-like ligands in the bone marrow. We found that all EGF-like ligands are potent chemoattractants for mesenchymal progenitors in vitro. EGF-like ligands are also known chemoattractants for other cells such as intestinal epithelial cells [46] and breast cancer cells [47], [48]. Loss of EGFR activity using an inhibitor, a neutralizing antibody, or siRNAs for EGFR significantly decreased the migration of mesenchymal progenitors toward PTH-treated conditioned media, providing direct evidence that activation of EGFR is involved in PTH-induced mesenchymal progenitor migration. Conditioned media from PTH-treated osteoblasts also stimulated the phosphorylation of EGFR, demonstrating that the EGFR was activated by a factor(s) released by osteoblasts. Furthermore, knockdown of amphiregulin expression in osteoblasts abolished the increased migration of mesenchymal progenitors toward the PTH-treated conditioned media, suggesting that amphiregulin is critical for the migratory effect of PTH. However, knockdown of amphiregulin did not significantly affect the migration toward control conditioned media, suggesting that amphiregulin expression in the absence of PTH treatment may be too low to mediate osteoblast-simulated mesenchymal progenitor migration. Loss of PI3K/Akt and p38MAPK activity, two downstream signaling pathways of the EGFR, also inhibited migration toward PTH-treated conditioned media. These pathways have been previously shown to play a role in EGF-stimulated motility of intestinal epithelial cells [49], [50]. We therefore propose that PTH stimulates mesenchymal progenitor migration indirectly through activation of the EGFR on mesenchymal progenitors and subsequent activation of the PI3K and p38MAPK signaling pathways.

Our data also suggest that EGFR activity in osteoprogenitors is required for a full anabolic response to PTH. We previously demonstrated that loss of EGFR in osteoprogenitors/osteoblasts (*Col-Cre Egfr^Wa5/flox^* mice) led to a significant decrease in EGFR activity in osteoprogenitors, great reduction in bone mass, and suppression of bone formation in skeletally mature mice [24]. Here, we demonstrate that these mice do not respond to PTH with an anabolic response in bone. A recent study reported normal anabolic actions of PTH on *Egfr^Wa5/+^* mice [38], which is consistent with our current findings. Although *Egfr^Wa5/+^* mice have low EGFR activity, we and other groups found that they exhibit similar bone phenotypes as wild-type mice [24], [37], [38]. We had to combine conditional *Egfr* knockout with the Wa5 mutation to achieve a more complete reduction in EGFR activity in order to observe significant bone phenotypes [24]. Therefore, we believe that the Wa5 mice still exhibit a significant bone anabolic response to PTH since they only have a moderate reduction in EGFR activity and a larger reduction in EGFR activity, such as what we have achieved with *Col-Cre Egfr^Wa5/flox^* mice, is required to demonstrate the effect of loss of EGFR activity on PTH treatment.

In summary, our data indicate that PTH stimulates osteoblasts and osteocytes to release amphiregulin which acts as a soluble chemotactic factor for mesenchymal progenitors. Together with our previous reports that activation of EGFR signaling stimulates the proliferation of mesenchymal progenitors but inhibits their differentiation, and our in vivo finding that the skeleton of EGFR-deficient mice does not respond to PTH injections, we propose that EGFR signaling plays an important role in mediating the anabolic response of bone to PTH treatment and therefore, it represents a new target for anabolic osteoporosis therapy.

## Supporting Information

Table S1 **Sequences of primers used for qRT-PCR.**
(DOC)
