# Supplementary material for: Amphiregulin-EGFR Signaling Mediates the Migration of Bone Marrow Mesenchymal Progenitors toward PTH-Stimulated Osteoblasts and Osteocytes
Source: PLoS One. 2012 Dec 31;7(12):e50099. doi: 10.1371/journal.pone.0050099 (PMC3534030; doi:10.1371/journal.pone.0050099)
Supplement: Table S1 — Sequences of primers used for qRT-PCR. (DOC) [file pone.0050099.s001.doc]

**Table S1**

| **gene** | **5’ primer** | **3’ primer** |
| --- | --- | --- |
| rat β-actin | TCC TGA GCG CAA GTA CTC TGT G | CGG ACT CAT CGT ACT CCT GCT T |
| rat GAPDH | AAC CCA TCA CCA TCT TCC AGG | GCC TTC TCC ATG GTG GTG AA |
| rat PTH1R | TGG ATG CGG ACG ATG TCT TT | TGC TGT GTG CAG AAC TTC CTT G |
| rat amphiregulin | CGA CCT ATC CAA GAT CGC GT | GAA TCG TTT CCG AAG CAG GA |
| human β-actin | AAG GAG AAG CTG TGC TAC GTC G | ACC GCT CAT TGC CAA TGG T |
| human EGFR | TCC CCG TAA TTA TGT GGT GAC A | AGG CCC TTC GCA CTTCTT ACA |
| mouse β-actin | TCC TCC TGA GCG CAA GTA CTC T | CGG ACT CAT CGT ACT CCT GCT T |
| mouse amphiregulin | TTT GGT GAA CGG TGT GGA GAA | CGA GGA TGA TGG CAG AGA CAA |
